# The Effect of Continuity of Care on Emergency Room Use for Diabetic Patients Varies by Disease Severity

**DOI:** 10.2188/jea.JE20150045

**Published:** 2016-08-05

**Authors:** Chia-Hsiang Hsu, Yiing-Jenq Chou, Christy Pu

**Affiliations:** Department of Public Health, National Yang-Ming University, Taipei, Taiwan ROC

**Keywords:** continuity of care, diabetes severity, comorbidity, emergency room use

## Abstract

**Background:**

Although many studies have reported that high-quality continuity of care (COC) is associated with improved patient outcomes for patients with diabetes, few studies have investigated whether this positive effect of COC depends on the level of diabetes severity.

**Methods:**

A total of 3781 newly diagnosed diabetic patients selected from the 2005 National Health Insurance database were evaluated for the period 2005–2011. Generalized estimating equations combined with negative binomial estimation were used to determine the influence of COC on the overall emergency room (ER) use and diabetes mellitus (DM)-specific ER use. Analyses were stratified according to diabetes severity (measured using the Diabetes Complications Severity Index [DCSI]), comorbidities (measured using the Charlson comorbidity score), and age.

**Results:**

COC effects varied according to diabetes severity. Stratified analysis showed that the positive effect of COC on DM-specific ER use was the highest for a DCSI of 0 (least severe), with an incidence rate ratio (IRR) of 0.49 (95% CI, 0.41–0.59) in the high-COC group (reference group: low-COC group). Compared with the low-COC group, high-quality COC had a significant beneficial effect on overall ER use in younger patients (IRR 0.51; 95% CI, 0.39–0.66 for the youngest [18–40 years] group, and IRR 0.67; 95% CI, 0.59–0.76 for the oldest [>65 years] group) and those with a high number of comorbidities.

**Conclusions:**

The positive effects of high-quality COC on the treatment outcomes of patient with diabetes, based on the overall and DM-specific ER use, depends on the level of disease severity. Therefore, providing health education to enhance high-quality COC when the disease severity is low may be critical for ensuring optimal positive effects during diabetes disease progression.

## INTRODUCTION

Continuity of care (COC) is a crucial aspect of family medicine^1^ and entails having a regular provider for disease treatment, thereby enhancing patient outcomes through care responsibility instead of managing specific conditions individually.^2^ Numerous studies have reported that high-quality COC is associated with improved outcomes in patients with diabetes. These positive patient outcomes include decreased all-cause mortality,^3^ decreased acute and avoidable hospitalization rates,^3^^–^^5^ improved weight control, reduced fasting blood glucose,^6^ and greater patient satisfaction.^7^ In addition, several studies have reported an association between high-quality COC and decreased emergency room (ER) use for patients with diabetes.^8^^–^^10^

However, previous studies have not determined whether there is an interaction effect between COC and diabetes severity on patient outcomes. There are several reasons to suspect that the interaction effects of COC on patient outcome may depend on diabetes severity, because the two effects may be correlated.^11^^,^^12^ COC is often associated with disease severity, and the treatment behavior of patients is also associated with disease severity. A meta-analysis study revealed that the objective severity of disease and patients’ awareness of this severity predicts the patients’ treatment behavior, such as adherence to a physician’s orders.^13^ This previous study reported that the main mechanism involved the efforts for enhancing care being disrupted by the stress of the disease, emotional distress, social isolation, and threat to identity.^13^ For patients with diabetes, the severity of comorbid conditions, such as depression, affects the adherence to crucial aspects of diabetes self-care.^14^

The positive effects of COC on patient outcomes are often due to patient satisfaction, which, in turn, is often associated with disease severity.^15^^,^^16^ Gill et al^17^ reported that, after controlling for diseases severity, no significant associations were observed between provider continuity and improved monitoring for patients with diabetes, indicating that the effects of COC may depend on disease severity.

A previous study found that patients anticipating the development of additional diabetes-related complications were more likely to exhibit deteriorated physical and mental functioning.^18^ Reportedly, patients with severe diabetes are more likely to expect such complications, such as vision impairment,^19^ than those with a mild condition, which may then disrupt the advantages of high-quality COC and result in poorer patient outcomes.

The present study aimed to determine the interaction effects of COC and diabetes severity on ER use in patients with DM. If the positive effects of COC depend on diabetes severity, providing health education to enhance high-quality COC when disease severity is low may be critical for ensuring optimal positive effects during diabetes disease progression. We hypothesized that high-quality COC is more effective in patients with lower diabetes severity because lower disease severity is less likely to disrupt the positive effects of COC on patient outcomes.

## METHODS

### Data and study sample

We used the Taiwan National Health Insurance (NHI) claims database (organized by the National Health Research Institutes of Taiwan) to include one million randomly selected insurants who enrolled in the NHI program in 2005 and who are representative of Taiwan’s population in 2005. The Taiwan NHI program is a public insurance system with compulsory enrollment of all residents in Taiwan. A person could be included in the sample despite the absence of any physician visits during that year. All insurants, regardless of their physician visits, were longitudinally followed from 2005 to 2011, and all claims data under the NHI were available. The medical claims under the NHI were sent to the National Health Insurance Administration of Taiwan for validation of the diagnosis coding. This study has been approved by the Institutional Review Board of National Yang-Ming University, Taiwan.

Type 2 diabetes was defined using the International Classification of Diseases, Ninth Revision, Clinical Modification (ICD-9 CM) as ICD-9-CM 250.xx. Patients who were newly diagnosed with diabetes in 2005 were included in the study sample. The three inclusion criteria for this study were: (1) the patient was not diagnosed with diabetes before 2005, to ensure that only newly diagnosed patients with diabetes were included; (2) the subject must have had at least 2 outpatient diabetes diagnoses, in addition to at least one prescription of an antidiabetic agent in 2005, or at least one inpatient use with the primary diagnosis being diabetes in 2005 along with use of an antidiabetic agent (this criterion was used to indicate a certain diagnosis of diabetes and was considered more accurate than using diabetes diagnosis alone); and (3) the patient was alive on December 31, 2011, to ensure that all the study patients were analyzed across a similar study period. The final criterion was essential because, when a patient died within a short period since the first diabetes diagnosis, the major causes of death may not have been diabetes. Moreover, such a patient group may have been already suffering from other complicated health concerns; thus, this criterion was used to ensure a homogeneous study sample.

Following patient selection based on these criteria, the annual COC for each patient was calculated. Overall, 3781 patients fulfilled the aforementioned criteria.

### Measures

#### Continuity of care

We used the COC index (COCI) to measure COC in this study. This index has been widely used in several previous studies.^21^^–^^23^ For each patient, COC was operationalized as described previously^24^:

$$\text{COC}=\left[\left(\sum_{i=1}^{p}n_{i}^{2}\right)-T\right]/T(T-1)$$

where *T* is the total number of DM-related outpatient visits; *n_i_* is the number of times the patient visited a physician *i*; and *p* is the total number of physicians visited. The index value ranged from 0 to 1, where 0 indicated *no continuity* and 1 indicated *perfect continuity*.

An outpatient visit was included in the COC calculation when the patient was diagnosed with ICD-9 CM 250.xx and was prescribed at least one antidiabetic agent during the same visit. We excluded inpatient visits since they may have been a COC outcome.^3^^,^^20^ We included only visits in which an antidiabetic agent was prescribed because a patient could have been diagnosed with diabetes at a visit that was unrelated to diabetes treatment. For instance, a physician may have simply noted that the patient had diabetes during a particular visit.

For a patient to receive a valid COC score for a particular year, a minimum of three diabetes outpatient visits was mandatory because COC remains invalid with a limited number of visits. Most studies have excluded patients with <3 visits.^9^^,^^21^ However, we included patients with <3 diabetes outpatient visits as a separate group (the “no-index group”). Excluding such patients could have biased the results because this patient group can exhibit unique characteristics. Moreover, because this patient group represented the largest group in our data, excluding this group from the analysis was inappropriate.

We separated the patients into four groups based on the COC level as follows: no index, low, medium, and high. The patients with COC = 1 (*perfect continuity*) were categorized into a separate “high” group because a high proportion of patients exhibited this characteristic. We categorized the remaining patients who did not belong to the “no-index” or “high” groups into two groups of approximately equal sizes (the number of patients between both groups may slightly vary based on the COC distribution). The patients with lower and higher COC scores were categorized into the “low” and “medium” groups, respectively, using a cut-off chosen to balance the groups.

#### Outcome variables

Two outcome variables were used in this study: overall ER use (ER visits with any diagnostic code) and DM-specific ER use (ER visits with the diagnostic code ICD9-CM-250.xx). Both ER use outcomes were defined as the number of times a patient visited the ER during the study period.

#### Other variables

##### Diabetes severity

The severity of diabetes was estimated using the Diabetes Complications Severity Index (DCSI), which is a measure of the number and type of diabetes complications. These complications include retinopathy, nephropathy, neuropathy, cerebrovascular disease, cardiovascular disease, peripheral vascular disease, and metabolic disease. The method used to construct this index has been described in detail elsewhere.^25^ The DCSI is considered an accurate indicator of diabetes severity and provides accurate predictions of mortality and risk of hospitalization in patients with diabetes.^25^ In addition, a previous study has demonstrated the accuracy of the DCSI regarding the assessment of type 2 diabetes severity using claims data.^26^

##### Comorbidity score

The Charlson comorbidity index (CCI) score, which was used to estimate comorbidity, is calculated based on 17 disease categories. A patient was considered to have a comorbid condition within 1 year if he or she had at least three claim records with an ICD-9 code for that condition during that particular year. A higher score indicates greater comorbidity.

##### Demographic and socioeconomic variables

Age, sex, area of residence, and socioeconomic status were included in the analysis. Socioeconomic status was determined based on insurance income. Fishermen and farmers do not have a clearly defined wage, so they were included in a separate group when the socioeconomic status variable was constructed.

### Statistical analysis

Because the no-index group was extremely different from the other COC groups and, by definition, lacked a valid COC measure, we excluded this group from the regression analysis. The COC group to which a patient belongs can vary from year to year. To account for the clustering effect caused by repeated measurements, a negative binominal model was estimated using generalized estimation equations. An exchangeable correlation structure was used. We stratified our analysis according to diabetes severity (DCSI score), comorbidity (Charlson comorbidity score), and age. Incidence rates (overall ER or DM-specific ER/person year) and incidence rate ratios (IRRs) are presented.

## RESULTS

Table 1 lists the sample characteristics observed during the selected years. In 2005, most patients belonged to the no-index group, followed by the high-COC group (perfect COC). The no-index group gradually decreased in size over the years, whereas the perfect continuity group increased in size. Regarding diabetes severity, most patients exhibited a DCSI score of zero, indicating no severe diabetes. However, as expected, this proportion declined over time.

**Table 1.** Sample characteristics by selected years (*n* = 3757)

	2005		2008		2011	
		
	*n*	%	*n*	%	*n*	%
Mean age, years						
20–39 (reference)	313	8.33	313	8.33	313	8.33
40–65	2385	63.48	2385	63.48	2385	63.48
>65	1059	28.19	1059	28.19	1059	28.19
Sex, male	1977	52.62				
Care continuity						
No index	1484	39.5	1079	28.72	1025	27.28
Low	467	12.43	525	13.97	559	14.88
Medium	485	12.91	594	15.81	545	14.51
High (COCI = 1)	1321	35.16	1559	41.5	1628	43
Income, New Taiwan $						
<20 000	1370	36.47	1035	27.55	1036	27.58
20 000–39 999	931	24.78	1221	32.5	1102	29.33
≥40 000	651	17.33	656	17.46	741	19.72
Fishermen/farmers, *n*	805	21.43	845	22.49	878	23.37
Area						
Taipei	1253	33.35	1248	33.22	1251	33.3
North	487	12.96	482	12.83	489	13.02
Central	682	18.15	690	18.37	691	18.39
South	578	15.38	587	15.62	576	15.33
Kaohsiung-Pingdong	651	17.33	652	17.35	651	17.33
East	106	2.82	98	2.61	99	2.64
Diabetes Complications Severity Index						
0	2674	71.17	2612	69.52	2322	61.8
1	647	17.22	669	17.81	780	20.76
≥2	436	11.61	476	12.67	655	17.43
Charlson Comorbidity Index						
0–1	2193	58.37	2209	58.8	1903	50.65
2	957	25.47	859	22.86	935	24.89
≥3	607	16.16	689	18.34	919	24.46
Frequency of emergency room use						
Mean/standard deviation	0.40/1.04		0.33/1.09		0.50/1.80	
Min	0		0		0	
Max	24		29		85	
Frequency of emergency room use, DM-related						
Mean/standard deviation	0.07/0.32		0.06/0.38		0.11/0.44	
Min	0		0		0	
Max	4		14		6	

COCI, Continuity of Care Index; DM, diabetes mellitus.

Chi-squared was used for categorical variables and ANOVA for continuous variables.

Unit of observation: individual patient.

1 United States $ ≈ 30 New Taiwan $.

Table 2 lists the incidence rates and IRRs estimated using a negative binomial model for clustered variance. Patients in the medium- and high-COC groups exhibited significantly lower IRRs for overall ER use than patients in the low-COC group did. The effect of the DCSI was statistically significant, with higher DCSI scores being associated with more frequent overall ER use. In addition, the age variable was significant in this model, and patients aged 40–65 years had lower IRRs compared with the reference group (18–40 years). However, the sex variable was nonsignificant. The variable of area of residence was marginally significant, and patients with higher income were less likely to visit the ER. Table 3 lists the results with DM-specific ER visits as the dependent variable. The direction of estimation was similar to that in the previous model.

**Table 2.** Negative binomial generalized estimation equation for the effect of continuity of care on overall emergency room use

	Incidence rate	Crude IRR	95% CI	Adjusted IRR	95% CI
Continuity of Care Index (COCI)					
Low (reference)	0.53				
Medium	0.44	0.86**	(0.80, 0.93)	0.89**	(0.82, 0.96)
High (COCI = 1)	0.30	0.61**	(0.57, 0.66)	0.67**	(0.62, 0.71)
Sex					
Male (reference)	0.37				
Female	0.38	1.01	(0.94, 1.10)	1.04	(0.96, 1.12)
Age, years					
20–39 (reference)	0.41				
40–65	0.34	0.83*	(0.71, 0.96)	0.74**	(0.64, 0.86)
>65	0.45	1.12	(0.96, 1.31)	0.86	(0.73, 1.00)
Area					
Taipei	0.38				
North	0.40	1.04	(0.92, 1.18)	0.98	(0.86, 1.11)
Central	0.39	1.00	(0.90, 1.12)	0.94	(0.84, 1.06)
South	0.34	0.91	(0.81, 1.03)	0.82**	(0.72, 0.93)
Kuoshong-Pingdong	0.34	0.88*	(0.78, 0.99)	0.84**	(0.75, 0.94)
East	0.45	1.12	(0.89, 1.41)	1.10	(0.87, 1.40)
Charlson Comorbidity Index					
1 (reference)	0.25				
2	0.39	1.50**	(1.40, 1.62)	1.36**	(1.26, 1.47)
≥3	0.67	2.51**	(2.33, 2.70)	1.97**	(1.81, 2.15)
Diabetes Complication Severity Index					
0 (reference)	0.29				
1	0.40	1.32**	(1.22, 1.42)	1.05	(0.97, 1.14)
≥2	0.74	2.42**	(2.24, 2.62)	1.67**	(1.52, 1.82)
Income, New Taiwan $					
<20 000 (reference)	0.43				
20 000–39 999	0.34	0.84**	(0.78, 0.92)	0.93	(0.85, 1.01)
≥40 000	0.32	0.82**	(0.74, 0.90)	0.87*	(0.79, 0.97)
Fishermen/farmers	0.39	0.96	(0.87, 1.06)	1.01	(0.91, 1.12)

CI, confidence interval; IRR, incidence rate ratio.

*P < 0.05.

**P < 0.01.

1 United States $ ≈ 30 New Taiwan $.

**Table 3.** Negative binomial generalized estimation equation for the effect of continuity of care on DM-specific emergency room use

	Incidence rate	Crude IRR	95% CI	Adjusted IRR	95% CI
Continuity of care index (COCI)					
Low (reference)	0.14				
Medium	0.11	0.85*	(0.74, 0.97)	0.86*	(0.75, 0.99)
High (COCI = 1)	0.06	0.50**	(0.44, 0.56)	0.54**	(0.47, 0.61)
Sex					
Male (reference)	0.09				
Female	0.09	0.94	(0.83, 1.08)	0.98	(0.86, 1.11)
Age, years					
20–39 (reference)	0.12				
40–65	0.08	0.64**	(0.51, 0.81)	0.56**	(0.45, 0.70)
>65	0.11	0.91	(0.71, 1.15)	0.65**	(0.51, 0.82)
Area					
Taipei	0.09				
North	0.10	1.05	(0.86, 1.30)	0.95	(0.77, 1.18)
Central	0.11	1.21*	(1.01, 1.44)	1.10	(0.92, 1.32)
South	0.08	0.92	(0.75, 1.13)	0.80*	(0.65, 0.99)
Kuoshong-Pingdong	0.08	0.84	(0.69, 1.03)	0.80*	(0.65, 0.97)
East	0.11	1.27	(0.88, 1.84)	1.23	(0.85, 1.78)
Charlson Comorbidity Index					
1 (reference)	0.05				
2	0.10	1.74**	(1.52, 2.00)	1.50**	(1.30, 1.73)
≥3	0.17	3.09**	(2.71, 3.52)	2.15**	(1.83, 2.52)
Diabetes Complication Severity Index					
0 (reference)	0.06				
1	0.09	1.37**	(1.19, 1.58)	1.05	(0.90, 1.23)
≥2	0.21	3.21**	(2.81, 3.65)	2.09**	(1.79, 2.44)
Income, New Taiwan $					
<20 000 (reference)	0.11				
20 000–39 999	0.08	0.75**	(0.65, 0.88)	0.86	(0.74, 1.00)
≥40 000	0.08	0.80*	(0.67, 0.96)	0.90	(0.75, 1.08)
Fishermen/farmers	0.09	0.93	(0.79, 1.10)	1.02	(0.86, 1.21)

CI, confidence interval; IRR, incidence rate ratio.

*P < 0.05.

**P < 0.01.

1 United States $ ≈ 30 New Taiwan $.

**Figure 1.**
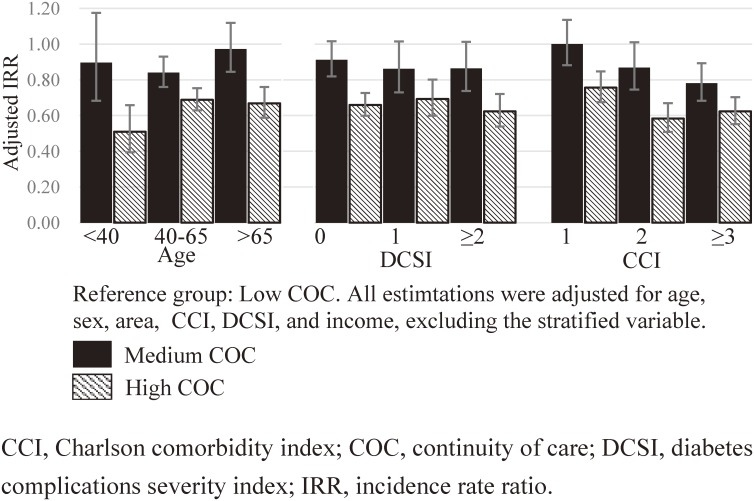
Adjusted IRRs for the effect of COC on total ER use, stratified by age, DCSI, and CCI.

**Figure 2.**
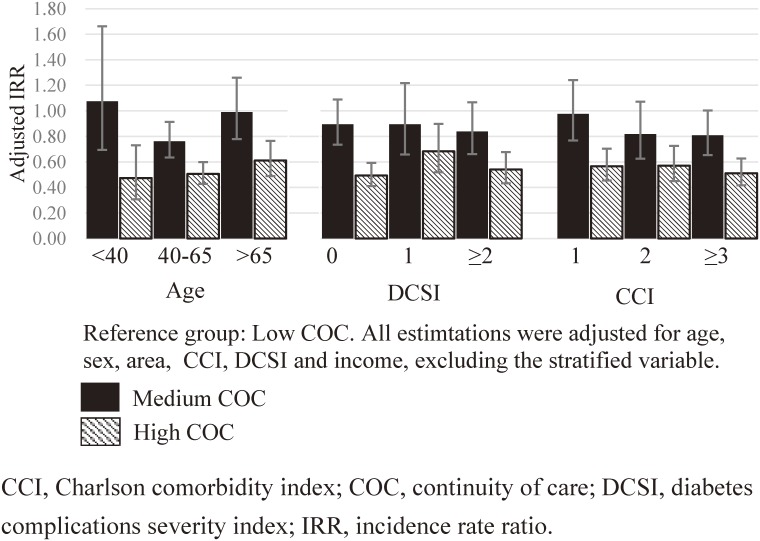
Adjusted IRRs for the effect of COC on DM-specific ER use, stratified by age, DCSI, and CCI.

We then stratified our analysis according to diabetes severity, comorbidity, and age. Figure 1 shows the adjusted IRR for COC. Each IRR pair (medium and high COC) was estimated using a separate model, with the reference group being the low-COC group. High COC helps reduce ER use in younger patients (Figure 1). In addition, high COC benefits patients with high CCI scores. The effect of COC did not vary according to diabetes severity for overall ER visits. However, for DM-specific ER visits (Figure 2), being in the high-COC group most benefitted those with DCSI = 0, followed by those with DCSI ≥2. Compared with the low-COC group, the medium-COC group had a significantly lower overall and DM-specific ER visits; however, this result was observed only in the age group of 40–65 years. This revealed different effects of COC when stratified according to age.

## DISCUSSION

This study investigated whether COC interacts with diabetes severity to affect the total number of overall ER and DM-specific ER visits. Many previous studies have reported that high-quality COC is associated with lower frequency of ER visits; however, to our knowledge, none of these studies has investigated whether this effect depends on disease severity. We demonstrated that the effects of COC in reducing DM-specific ER use were the strongest in patients with low diabetes severity.

Our finding that high-quality COC leads to improved patient outcomes for patients with diabetes is consistent with results reported by most previous studies; however, such positive effects are affected by disease severity. For example, the positive effects of high-quality COC decreased when the DCSI score was 1 but increased again when the DCSI score was ≥2. This suggests that health education to promote high-quality COC should be initiated at an earlier stage, when the disease is less severe.

A possible explanation is that, at a non-severe stage, patients can enjoy the positive outcomes because of less disruption from the disease itself and thus gain confidence in their physician, which causes further alterations in their behaviors, such as increased medication adherence.^21^ In addition, as disease severity increases, the treatment or self-management of disease may outweigh the importance of COC. Patients with type 2 diabetes are prescribed multiple medications to improve several aspects of their disease, such as metabolic control, reducing serum glucose and cholesterol levels, and blood pressure control.^27^ However, evidence suggests that the number of medications can affect patient behavior, such as medication adherence.^28^ Thus, when patients with high disease severity are prescribed an increased number of medications, the beneficial effects of COC may be disrupted because of the differences in treatment and patient behavior. However, patients with high diabetes severity may already be familiar with the treatment and therefore may benefit from the effects of COC. When the disease progresses from the mild to medium stage, the patient’s perception of the illness changes. Such perceptions often affect their coping strategies, including self-management behaviors,^27^ making it more challenging to manage severe diabetes thereby disrupting the beneficial effects of COC.

Furthermore, the effect of COC varied according to age, revealing that COC is more beneficial for younger patients than for older patients with diabetes. One reason could be that, in patients newly diagnosed with diabetes, younger patients are more concerned than older patients are because it may signal the initiation of a decline in their health status, and this concern prompts them to engage in better disease management. This may cause high-quality COC to be more effective for younger patients.

We found that COC is more beneficial for patients with more comorbidities. An explanation may be that, among patients newly diagnosed with diabetes, patients with a high number of comorbidities possess substantial knowledge about chronic disease management and can therefore obtain greater benefits from high-quality COC. Furthermore, this may imply that, for patients newly diagnosed with diabetes, health education related to disease management may be equally important to promoting high-quality COC.

This study has certain limitations. First, we did not determine causal effects because diabetes severity is a patient outcome; as such, severity can act as a dependent variable rather than an independent one. In addition, the disease condition of patients with unsatisfactory self-management behavior may have been more severe than in those with effective management, and such patients may have practiced lower COC because some their personal traits may not have been accurately captured by the variables in this study. Thus, our results should be interpreted only as an association. Second, although the two widely used patient outcome measures are consistent with those used in previous studies (overall ER use and DM-specific ER use), these outcome variables do not capture all patient outcomes for all patients with diabetes. For instance, blood-glucose control may be a more important patient outcome in patients with diabetes than either of the outcomes used in our study. However, our claims data provided no such information. We selected the current dependent variables because they were available and have been widely used by previous studies using claims data.^8^^,^^9^^,^^29^^,^^30^

In conclusion, the positive effect of high-quality COC varies according to diabetes disease severity level, and this effect should be evaluated while accounting for other comorbidities in patients with diabetes. In addition, the effects of COC vary according to age. Regarding diabetes care, the promotion of high-quality COC should be initiated early after disease onset.
